# Comprehensive Genome Analysis of Carbapenem-Resistant Strains of *Raoultella* Species, an Emerging Multidrug-Resistant Bacterium in Hospitals

**DOI:** 10.1128/AAC.01367-19

**Published:** 2019-11-21

**Authors:** Xiao Yu, Beiwen Zheng, Jing Zhang, Hao Xu, Tingting Xiao, Yanzi Zhou, Shuntian Zhang, Yunying Zhu, Yuan Wang, Yonghong Xiao

**Affiliations:** a State Key Laboratory for Diagnosis and Treatment of Infectious Diseases, National Clinical Research Center for Infectious Diseases, Collaborative Innovation Center for Diagnosis and Treatment of Infectious Diseases, The First Affiliated Hospital, College of Medicine, Zhejiang University, Hangzhou, China; b Department of Respiratory Diseases, The First Affiliated Hospital, College of Medicine, Zhejiang University, Hangzhou, China

**Keywords:** *Raoultella* spp., carbapenem resistance, mobile genomic elements, transposons, mechanisms of resistance, molecular epidemiology

## Abstract

We report the characterization of six carbapenem-resistant *Raoultella* spp. (CRRS) in our hospital and a genomic analysis of 58 publicly available isolates. CRRS isolates are sporadically identified around the world, and different transposons carrying carbapenemases were the resistant mechanisms. Mobile genetic elements play an important role in acquiring antibiotic resistance genes from the hospital. An improved understanding of these transposon and targeted control measures will be very valuable to prevent CRRS dissemination.

## Abstract (precis)

We report the characterization of six carbapenem-resistant *Raoultella* spp. (CRRS) in our hospital and a genomic analysis of 58 publicly available isolates. CRRS isolates are sporadically identified around the world, and different transposons carrying carbapenemases were the resistant mechanisms. Mobile genetic elements play an important role in acquiring antibiotic resistance genes from the hospital.

## TEXT

*Raoultella* species, which were moved into a separate genus from genus *Klebsiella* in 2001, are normally found in aquatic environments (1). Due to the introduction of accurate molecular techniques in clinical microbiology laboratories, there has been an increasing trend in the number of reports of clinical infections with *Raoultella* species strains (2). Notably, cases of infection with carbapenem-resistant *Raoultella* spp. (CRRS) have been sporadically reported (3–7). In recent years, despite the high mortality in clinical infection, there has not yet been a systematic study of the mechanisms underlying carbapenem resistance in *Raoultella* species strains (8).

### Characteristics of *Raoultella* species isolates.

**FIG 1:**
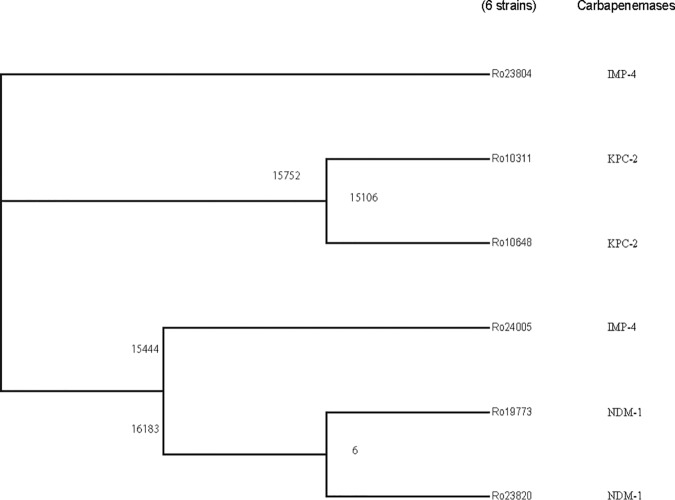
Phylogeny and carbapenemases of six carbapenem-resistant *R. ornithinolytica* strains. The tree on the left was constructed based on core genome SNPs. The second column shows the carbapenemases carried by these strains. The numbers in the figure indicates the number of SNPs: 15,752 SNPs between Ro23804 and Ro10311, 15,106 SNPs between Ro10648 and Ro10311, 15,444 SNPs between Ro23804 and Ro24005, 16,183 SNPs between Ro23804 and Ro19773, and 6 SNPs between Ro19773 and Ro23820.

From January 2013 to December 2016, six CRRS strains were isolated in our hospital. The drug sensitivities of the CRRS are shown in Table 1. Whole-genome sequencing of these strains showed three different carbapenemase genes, including *bla*_IMP-4_ (Ro23804 and Ro24005), *bla*_KPC-2_ (Ro10311 and Ro10648), and *bla*_NDM-1_ (Ro19773 and Ro23820). The phylogenetic results of the six CRRS strains show that strains carrying the same carbapenem resistance genes cluster closely. According to the date of isolation, the 3 couples of CRRS strains appeared to be compatible with cross-transmission; however, the number of single nucleotide polymorphisms (SNPs) from the core genome does not support this speculation (Fig. 1).

**TABLE 1:** Characteristics and antimicrobial resistance of isolates and transconjugants

Strain ID^*a*^	Specimen	Strain	Isolation date (yr-mo-day)	MIC (μg/ml)^*b*^													
				AMC	FEP	TZP	CAZ	CTX	AZM	IPM	MEM	GEN	AMK	CIP	LEV	SXT	TGC
Ro10311	Sputum	*R. ornithinolytica*	2013-05-06	>32/16	>64	>128/4	>64	>64	>64	>16	>8	<1	<2	1	1	<20	<0.5
Ro10648	Sputum	*R. ornithinolytica*	2013-05-20	>32/16	>64	>128/4	>64	>64	>64	>16	>8	<1	<2	1	1	<20	<0.5
Ro19773	Sputum	*R. ornithinolytica*	2014-06-25	>32/16	>64	>128/4	>64	>64	>64	>16	>8	<1	<2	>4	8	>320	<0.5
Ro23804	Sputum	*R. ornithinolytica*	2014-07-11	>32/16	>64	>128/4	>64	>64	>64	>16	>8	>16	>64	>4	2	<20	<0.5
Ro23820	Feces	*R. ornithinolytica*	2014-07-12	>32/16	>64	>128/4	>64	>64	>64	>16	>8	<1	<2	>4	8	>320	<0.5
Ro24005	Drainage	*R. ornithinolytica*	2014-08-21	>32/16	>64	>128/4	>64	>64	>64	>16	>8	>16	>64	1	1	<20	<0.5
J53-Ro19773	NA^*c*^	E. coli	NA	>32/16	4	>128/4	>64	>64	>64	>16	>8	0.5	<2	<0.25	<0.25	<0.5	<0.25
J53-Ro23820	NA	E. coli	NA	>32/16	4	>128/4	>64	>64	>64	>16	>8	0.5	<2	<0.25	<0.25	<0.5	<0.25

a ID, identifier.

b AMC, amoxicillin-clavulanic acid; AMK, amikacin; AZM, aztreonam; CAZ, ceftazidime; CIP, ciprofloxacin; CTX, ceftriaxone; FEP, cefepime; GEN, gentamicin; IPM, imipenem; LEV, levofloxacin; MEM, meropenem; SXT, trimethoprim-sulfamethoxazole; TGC, tigecycline; TZP, piperacillin-tazobactam.

c NA, not available.

**FIG 2:**
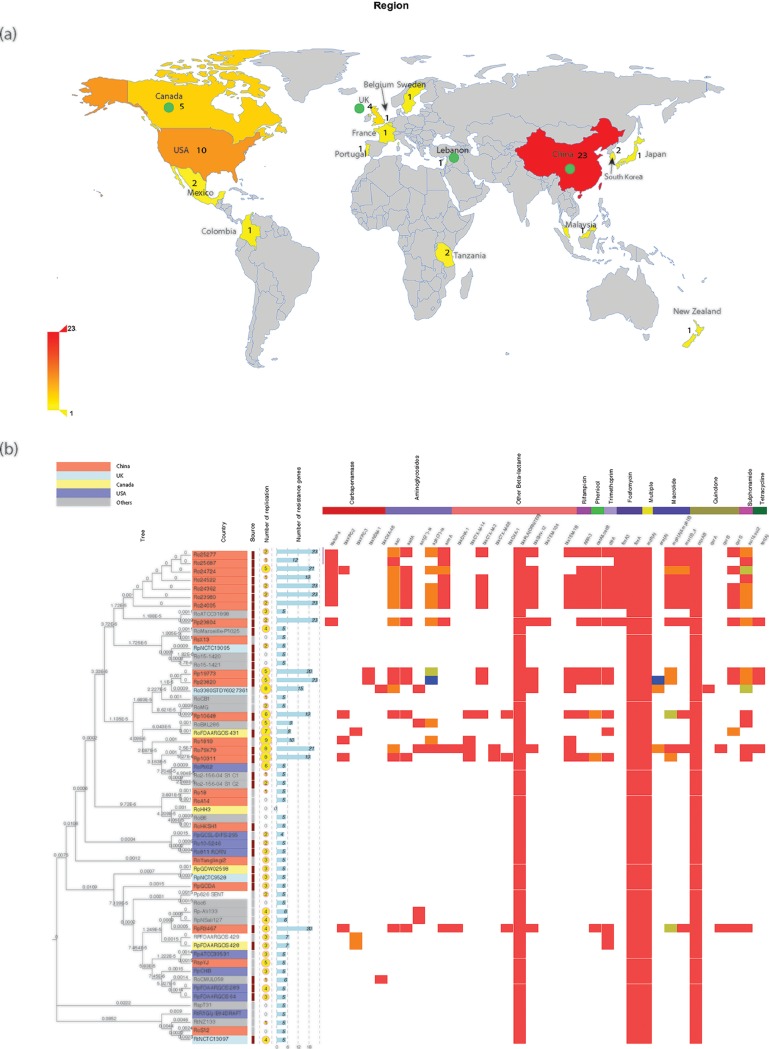
Distribution and characteristics of 58 *Raoultella* spp. with publicly available genomic data. (a) Distribution of *Raoultella* species isolates in the world; the numbers in the figure represent the numbers of isolated strains. Green circles represent the countries where CRRS strains are isolated. (b) The maximum likelihood phylogeny is shown on the left. The tree is drawn to scale, with branch lengths measured in the number of substitutions per site. There were a total of 543,877 positions in the final data set. Different background colors represent different countries. In the source column, solid red lines indicate that the strain was isolated from a hospital, and dotted lines indicate environment strains. The fourth column gives the number of plasmid replicons in each strain, and the fifth column shows the number of resistance genes. The heatmap indicates the presence of individual antimicrobial resistance, and the top part shows the kinds of drug resistance genes. It is noteworthy that strains Ro23980, Ro24005, Ro24362, Ro24724, Ro25277, and Ro25687 in this figure were isolated from the same patient.

Furthermore, a comparative genomics analysis of 58 *Raoultella* species strains whose genome sequences were publicly available (as of 30 September 2018) was performed. The basic information for the strains is provided in Fig. 2a and Table S1 in the supplemental material. In addition to the CRRS in our hospital, there were seven other CRRS strains, including two KPC-2-producing strains, three KPC-3-producing isolates, and two strains carrying *bla*_OXA-48_ (Fig. 2b). Five different carbapenemases were found in the 13 CRRS strains, suggesting no clear epidemic domain carbapenemases. In addition, the distribution of carbapenemases in CRRS isolates is consistent with the global distribution of carbapenemases, suggesting that carbapenem resistance genes are regionally distributed.

The S1-pulsed-field gel electrophoresis (PFGE) showed that the six CRRS strains carried different numbers of plasmids. Southern hybridization results showed that even the same carbapenemase is located on different-sized plasmids (see Fig. S1). The *bla*_NDM-1_ gene was successfully transferred to Escherichia coli J53 via conjugation, with a conjugation efficiency of 0.5 × 10^−5^. The drug sensitivities of the transconjugants are shown in Table 1. Conjugation experiments with strains carrying *bla*_KPC-2_ and *bla*_IMP-4_ were unsuccessful. We also tried to transfer plasmids extracted from isolates by chemical and electrical transformation. However, repeated transformation methods failed to move the plasmids to recipient E. coli DH5α cells. This may have primarily been due to the large size of the plasmids, which may limit the efficiency of transformation.

### Carbapenemase gene environments.

**FIG 3:**
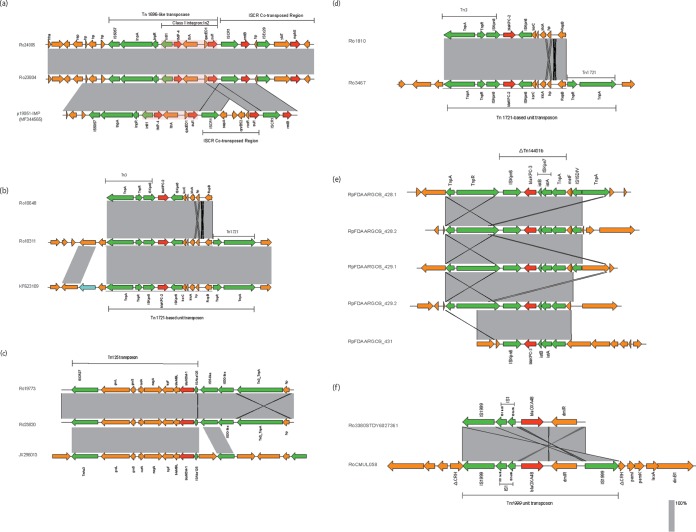
Schematic representations of the genetic organization surrounding carbapenemase resistance genes and comparisons with a closely related genetic structure. The gray regions between the plasmids indicate the nucleotide identity (85% to 100%) obtained from BLASTN. The arrows indicate predicted open reading frames (ORFs) whose names are given above the arrows. Green indicates genes related to mobile elements, and red indicates genes related to drug resistance. Orange represents other functional genes. (a) Major structural features of the *bla*_IMP-4_ gene regions in Ro24005 and Ro23804 in comparison with plasmid p19051-IMP (GenBank no. MF344565). (b) Schematic representations of the genetic organization surrounding the *bla*_KPC-2_ gene in Ro10311 and Ro10648 in comparison with plasmid pJM45 (GenBank no. KF623109). (c) Major structural features of the *bla*_NDM-1_ regions in Ro19773 and Ro23820 in comparison with the closely related pBJ01 plasmid (GenBank no. JX296013). (d) Major structural features of the *bla*_IMP-4_ gene regions in Ro1810 and Ro3467. (e) Major structural features of the *bla*_KPC-3_ regions in RpFDAARGOS_428.1, RpFDAARGOS_429.1, RpFDAARGOS_431, RpFDAARGOS_428.2, and RpFDAARGOS_429.2 isolates carried two different plasmids containing *bla*_KPC-3_ genes, respectively. (f) Major structural features of the *bla*_OXA-48_ regions in Ro3380STDY6027361 and RoCMUL058.

The analysis showed that the *bla*_IMP-4_ gene environments of Ro24005 and Ro23804 were the same, and this environment was similar to the structure of plasmid p19051-IMP (MF344565) which was carried by a Klebsiella pneumoniae strain isolated in Ningbo, China, except for an insertion sequence common region (ISCR) element (Fig. 3a). The *bla*_IMP-4_ gene is located in a complete class I integron In2, and this integron is inserted into a Tn*1696*-like transposon bounded by IS*5075* elements. A special insertion sequence, ISCR, which is similar to the IS*91* family transposons, lies downstream of this transposon. In this study, insertion of this integron constituted the main structure of a Tn*1696*-like transposon, which is the main transposon involved in *bla*_IMP-4_ dissemination in China (9). Therefore, we speculate that this transposon will lead to broad *bla*_IMP-4_ dissemination in the future. In addition, the class I integron In2 can capture drug resistance genes, and one or more ISCR sequences carrying multiple drug resistance genes are often inserted into the sequence downstream of the transposon (Fig. 3a, inserts show two ISCR sequences), which further increases the resistance of the strain. Since Tn*1696*-like transposons belong to the Tn*3* transposon family which are commonly reported in K. pneumoniae and Enterobacter cloacae (10), carbapenemases in CRRS may be obtained from these strains via transposon transposition. In the future, we must pay more attention to the role of Tn*1696*-like transposons in the transmission of *bla*_IMP-4_ carbapenem genes.

The *bla*_KPC-2_ gene is located between IS*Kpn8* and IS*Kpn6* insertion sequences. This region, “IS*Kpn8-bla*_KPC-2_-IS*Kpn6*,” is inserted downstream of the Tn*3* transposase and forms a Tn*1721*-based transposon that includes the *korC* and *klcA* genes (Fig. 3b). It is demonstrated that the Tn*1721*-based transposon is the main *bla*_KPC-2_-carrying transposon in *Enterobacteriaceae* bacteria (11, 12) and can mediate the transfer of the *bla*_KPC-2_ gene between different bacteria (12). Therefore, Tn*1721* may be further disseminated to other strains, including *Raoultella* spp., in the future. Interestingly, as show in Fig. 1, IMP-4-producing strains clustered closer to KPC-2-producing strains, possibly because these two carbapenemase genes were located on similar mobile elements, i.e., Tn*3* family transposons.

The annotation of the *bla*_NDM-1_ gene environment is based on that of JX296013, which was the first NDM-1 carbapenemase-producing E. coli strain isolated in Beijing. The *bla*_NDM-1_ gene is located between the IS*Aba125* and *ble*MBL genes. The main structure of the Tn*125* transposon consists of the insertion of the “IS*Aba125-bla*_NDM-1_-bleMBL region” and the “groL-groS-cutA-nagA region” downstream of IS*CR27* in CRRS strains. The IS*Aba125-bla*_NDM-1_-*ble*MBL gene structure is relatively stable in the Tn*125* transposon (Fig. 3c). Currently, almost all of the globally reported *bla*_NDM-1_ genes exist in this structure, and the Tn*125* transposon is often located on a conjugable plasmid, further leading to easy transmission of the *bla*_NDM-1_ gene (13, 14). It is thought that Tn*125* originated in Acinetobacter baumannii and was disseminated in this strain (15). Later, because of an insertion of a mobile element sequence in the initial segment of this transposon, e.g., the insertion of an ISCR sequence, the resulting novel Tn*125* transposon was mainly disseminated in *Enterobacteriaceae*, especially K. pneumoniae and E. coli (16). Because the structure of the Tn*125* in the *Raoultella* spp. was similar to this new type of Tn*125*, the *Raoultella* spp. may have acquired the resistance gene from *Enterobacteriaceae* family isolates.

Carbapenem gene structures of the seven publicly available CRRS strains were also explored. The two *bla*_KPC-2_ genes in one Raoultella ornithinolytica and one Raoultella planticola isolated from China is similar to Ro10311 found in our hospital, located on the Tn*1721*-based transposon (Fig. 3d). The *bla*_KPC-3_ gene in two *R. planticola* and one *R. ornithinolytica* isolate from Canada was located in the Tn*4401b* transposon with the *TnpR* gene replaced by IS*15DIV* (Fig. 3e). The *bla*_OXA-48_ in *R. ornithinolytica* (one from Lebanon and one from the United Kingdom) was located on the Tn*1999* transposon as reported by Poirel et al. (17). Downstream of *bla*_OXA-48_ was a *dmlR* gene encoding an HTH-type transcriptional regulatory protein. The Tn*1999* transposon inserted into the *CRH* gene, suggesting that this gene could possibly be a hot spot for integrating the Tn1*999* transposon (Fig. 3f).

The analysis of the carbapenemase gene environments showed that these carbapenem resistance genes were located on transposons. The same carbapenemases were located in the same transposon structure, suggesting that transposons play an important role in the dissemination of carbapenem resistance genes. In any case, due to the low clinical isolation rate of *Raoultella* species isolates, it is therefore very likely that these mobile elements were acquired from other strains in the *Enterobacteriaceae* family. Thus, this possible outcome requires significant attention and the implementation of targeted control measures.

### Genomic characteristics and drug resistance genes.

After removing repetitive strains, 51 of 58 *Raoultella* species strains were included for further analysis. The core genome of the 51 strains consisted of 1,627 genes and accounted for 29.99% (1,627/5,424) of the average total genome size of the various *Raoultella* spp. The relationship between the number of strains and the numbers of core genes or total genes showed that the *Raoultella* species strains contained an open pan-genome. Of the 51 strains, 20 were isolated from the environment and only one was a CRRS strain, while there were 10 CRRS in 31 strains which were isolated from hospitals. The carbapenem resistance rate in the hospital-isolated strains was significantly higher than that in the environmental strains (chi-square statistics, *P* = 0.009). This observation suggests that hospitals are the main source of carbapenem resistance genes in *Raoultella* spp. The average number of drug resistance genes isolated from hospital strains was 11.78, while only 5.35 were found in the environmental strains (*P* < 0.05). This difference indicates that the hospitals might have been the source of the drug resistance genes in these *Raoultella* species isolates. In addition, the average numbers of plasmid replicons carried by the hospital and environmental isolates were 3.13 and 2.08, respectively (Fig. 2b), suggesting that the main strategy for *Raoultella* spp. to acquire drug resistance genes in hospitals is via plasmid-mediated horizontal transfer.

Currently, most of the known genes that mediate carbapenem resistance in *Raoultella* species isolates are carried on plasmids, and we have observed the dynamic process of the plasmid-mediated acquisition of a KPC-2 gene in an *R. ornithinolytica* isolate *in vivo* (3, 5–7). In this study, we found that two NDM-1-producing *R. ornithinolytica* isolates were recovered from different samples (sputum and feces) from the same patient. Interestingly, the number and size of the plasmids carried by these two strains differed (Fig. S1, lanes 5 and 6). This observation further reflects the variability of the *Raoultella* spp. genome. Therefore, combined with our findings, these data further indicate that *Raoultella* spp. have an open pan-genome and that they can easily acquire exogenous genes, such as various kinds of antibiotic resistance genes shown in Fig. 2b.

For the first time, we comprehensively analyzed the *Raoultella* species and showed carbapenemases in CRRS strains correspond with the prevalent carbapenemases in the isolated region; a variety of transposons carrying different carbapenemases is the main mechanism for CRRS. The open pan-genome of *Raoultella* spp. may be associated with acquisition of drug resistance genes in hospitals via mobile genetic elements. An improved understanding of these transposon and targeted control measures will be very valuable to prevent the dissemination of CRRS.

## Supplementary Material

Supplemental file 1

Supplemental file 2
