# Supplementary material for: Comprehensive Genome Analysis of Carbapenem-Resistant Strains of Raoultella Species, an Emerging Multidrug-Resistant Bacterium in Hospitals
Source: Antimicrob Agents Chemother. 2019 Nov 21;63(12):e01367-19. doi: 10.1128/AAC.01367-19 (PMC6879265; doi:10.1128/AAC.01367-19)
Supplement: Supplemental file 1 [file AAC.01367-19-s0001.pdf]

Supplemental materials1

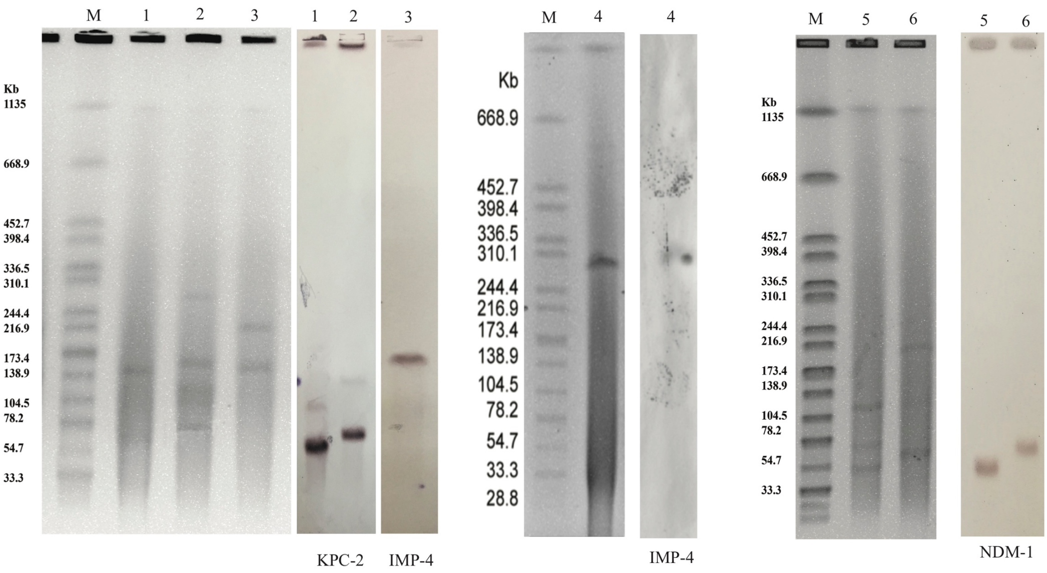

Figure S1. The results of S1-PFGE and Southern blot hybridization of six carbapenem-resistant R. ornithinolyticus strains. M: Marker; 1: Ro10311; 2: Ro10648; 3: Ro23820; 4: Ro24005; 5: Ro19773; and 6: Ro23820. Note: We previously reported that the IMP-4 gene carried by Ro24005 is located on the 300 kb plasmid.
